# The Development of the Pulp-Nodule

**Published:** 1901-10

**Authors:** 


The Development of the Pulp-Nodule. By D. E. Caush, L.D.S.I.[1]

Among the many and varied factors connected with the development of hard tissues, there is probably none more interesting than that of the development of secondary dentine; and in the development of the latter there is one question that has often puzzled the student as well as the more advanced thinker, and that is, Why is the microscopic structure of the pulp-nodule usually so different from that of secondary dentine?

In our endeavor to answer this interesting question we will commence by making a microscopic examination of a tooth in which we have diagnosed pulp-nodules, and make a section with the hard and soft tissue combined. We shall at once see a great difference between such a section and one of the tooth in which secondary dentine has been formed, whether for the purpose of resisting the attack upon the pulp as caused by decay, softening of the dentine, or where erosion has had the opportunity of leaving its permanent marks; or if we examine the section of a tooth worn down by friction resulting from mastication or attrition.

In all these cases we usually have a more or less pronounced deposit of secondary dentine, either in the pulp-chamber or in the pulp-canals. This new deposit is always caused by more or

[1] The original paper was very beautifully illustrated, confirming the author's views, but the text is sufficiently clear without their reproduction.—Ed.

less pronounced irritation from the *exterior* of the tooth or pulp, and the microscopic character of the new tissue corresponds usually to the structure as found in normal dentine, having tubuli often corresponding and associated directly with the tubuli of the dentine.

It would appear as if this new tissue had been Nature's method of defending herself from the attacks of the enemy from without, as the new tissue has been deposited by those cells surrounding the pulp itself, whose duty it is to protect the delicate pulp with its ramification of nerves, blood-vessels, etc., from external irritation; but in the development of the nodules of bone in the pulp this is not the case; the condition of the dentine does not appear to be a factor in the development of these nodules, as the irritation which eventually produces the pulp-nodule is produced *in the pulp-tissue* itself.

On examining a tooth there is nothing in the external appearance to suggest the existence of even the smallest of pulp-stones, nor can we take into consideration as a factor the age or sex of patients in the development of this tissue, as these nodules are to be found in the teeth of patients in their teens as well as in those of advanced age, and in many cases producing as much pain in the youngest as in those of old age; the result of my examinations tends to show that the position of the nodule is usually the cause of the pain produced.

In external appearance the nodules vary from a small, more or less globular, or oval, structure to that of any size or shape, controlled only by the size of that portion of the canal or chamber in which they are found. Thus, we have them from a minute point to those entirely filling the pulp-chamber, and in teeth of two or more roots it is not unusual to find them not only filling the pulp-chamber, but with spines of hard tissue passing into the various canals, and thus producing the most irregular-shaped nodules.

Their position is almost as varied as their outline, for, as I have already said, we may find them in the pulp-canals and pulp-chambers, quite free from the surrounding dentine, or we may find them attached to the sides of the pulp-canals, and quite surrounded by the dentine; we may, as I have said, find them anywhere in the pulp itself, from the entrance of the pulp at the apex of the root to the extreme end of the pulp-chamber. It is not the largest of these that cause the greatest amount of pain, as the pain is produced by the position and not by the size of the nodule; thus a small nodule near the apex of the canal will probably produce pain by the constriction of the pulp and, as a consequence, pressure upon the nerves, whilst a nodule much larger in size (unless there is pressure produced upon the nerves) may almost entirely fill the pulp-chamber without producing any discomfort. Though their size, shape, and position may vary very much, such is not the case with regard to their microscopic structure. All the nodules I have examined have a somewhat similar structure when viewed by the microscope. We have usually in the centre, or somewhere near the centre, of the developed tissue a space, more or less pronounced, and it is at this point that the nodule has its origin; it grows outwardly, and radiating from this point there are usually a number of more or less concentric rings caused by additional layers of calcified tissue; it is in this way a nodule increases in size; this may continue until two or more nodules touch each other and become united into one, thus forming a compound nodule large and irregular in shape. Its structure, as thus seen, is quite different from either ordinary or secondary dentine; we may sometimes find a few isolated irregular tubuli, but rarely do we find any tubuli approaching the character of those seen in secondary dentine; this may be accounted for by the fact that its origin is generally some distance from the odontoblastic layer, and the nodule is developed from a different layer of cells from that of secondary dentine. Its position would imply that no odontoblasts had taken part in its development, and if this is so, a very interesting question arises as to how these tubuli, if they are tubuli, are produced.

The origin of the development of the nodule is some irritation *in the pulp itself,* and I believe the primary object of the nodule is to cover up, by calcification, some substance that has been the cause of irritation in the pulp-tissue; its structure as well as its mode of development would lend itself to this supposition, for under the microscope we have a structure similar in appearance, and I believe identical in its mode of development, to that of the pearl found in the oyster, the origin of the pearl being a foreign body found in the mantle of the mollusc. As it is impossible for the oyster to get rid of this foreign body by absorption, it builds around this cause of irritation that which is known to us as the pearl. So in the development of the pulp-nodule the same has occurred: Nature has found in that delicate and complicated structure, the pulp, something it cannot get rid of, something it cannot absorb, something out of its place, and the irritation produced by this something causes the pulp to endeavor to get rid of it; as, however, it cannot absorb it, there is nothing for it to do but to encyst it.

If this is the true origin of the pulp-nodule, we ought to be able to find in some pulps, as a result of microscopic examination, the exciting cause, and this exciting cause is, I believe, to be found in either a dead cell or cells, or perhaps a few blood-corpuscles that have by some means escaped from their ordinary course, either by the rupture of one of the capillaries or small blood-vessels abounding in the pulp; such an accident may occur by a sudden shock to the tooth, or, again, the corpuscles may be found out of their place as a result of the breaking down of some of the smaller arteries, veins, or even the capillaries, by disease such as the pulp is exposed to.

I think the former method is the one that usually causes the development of pulp-nodules in our younger patients, whilst undoubtedly the changes in the pulp lend themselves to the development of these nodules as age advances. We will suppose, from either of the above, or some similar cause, that such a change has taken place in the pulp, and as a result there will be irritation of a more or less pronounced character in this tissue, produced by what has become a foreign body; there are no lymphatics for the reabsorption of this body, it does not require a very great stretch of the imagination to trace the growth of the pulp-nodule on the lines laid down. The course followed would, I believe, be somewhat as follows:

The irritation produced by the foreign body causes an increased activity in the blood-vessels surrounding the cause of the irritation, and, as a necessary consequence, an increase of formative material brought to hand. This material is taken out of the blood by the surrounding cells, and it becomes a very simple matter for these cells to cover up the cause of the irritation by a deposition of hard tissue. This probably occurs as follows: The increased blood-supply, produced by the irritation, causes immediate activity in the cells surrounding the cause of irritation, and the result of the activity is to produce a number of new cells. The pressure produced by this increase in the number of cells again increases the blood-supply, and after a time the cells deposit a hard tissue in the same manner as the cementum is produced. As the deposition takes place in very small quantities, and probably very slowly, we have a more or less perfectly calcified and, as a consequence, homogeneous deposition. This accounts for the structureless character of the nodule.

Again, we sometimes have a larger mass more rapidly formed, and perhaps nearer, or even including some of the odontoblasts in the newly forming mass. We shall then have variations in the structure, consisting of lacunæ with canaliculi, irregular spaces, and a few markings like the tubuli of dentine. As the calcification of these cells continues, we shall have an increase in the size of the nodule; in some cases this increase in size causes a further irritation of the surrounding pulp-tissue, and thus a constant supply of fresh formative material is brought to the cells. The increase of size eventually produces pressure upon the nerves of the pulp, and, as a consequence, frequently the most acute pain is experienced; this is the course, I believe, of the pulp-nodule when surrounded by pulp-tissue.

There is yet another class of nodule: though previously mentioned, it may be interesting to briefly follow its history. I refer to those nodules attached to the dentine. We may also, if we are fairly successful in our search, find them not only attached at one side, but entirely surrounded by dentine, and that at some little distance from the pulp-canal; wherever I have found these they have always been near the apex of the root and embedded in the last formed dentine; and this, I think, gives the key to the explanation of the position in which the nodules are found.

At the time the cutting edge of the tooth is passing through the gums the apex of the root is in an uncalcified condition, and with a new tooth in this position it is much more subject to a shock than it will be after it is fully erupted; as a consequence, we have a nodule formed in precisely the same manner as those formed in the pulp-tissue, with this exception: those found in the pulp-tissue are always formed *after* the calcification of the dentine has taken place, whilst the latter class are formed *prior* to the calcification of the tooth, and when first formed are surrounded by uncalcified dentine. Any careful examination of a tooth with nodules in this position will fully illustrate my meaning, as the tubuli of the dentine will be found to be bent around the nodule, proving the nodule *must* have been formed prior to the calcification of the dentine. If it had been otherwise, and the dentine had been absorbed to make a space for the nodule, we should then have found the tubuli ending abruptly at or near the margin of the nodule, but this is not the case.

This will also account for nodules found in the pulp-canals but attached to the dentine, and here, I believe, the development has been the same as in nodules surrounded by dentine.—*Transactions of Odontological Society of Great Britain.*

